# Rituximab monotherapy for splenic marginal zone lymphoma with villous lymphocytes: report on long-term disease control for two patients with recurrence after splenectomy

**DOI:** 10.1590/S1516-31802010000600012

**Published:** 2010-12-02

**Authors:** Márcio Debiasi, Marluce Hehnemann, Bernardo Garicochea

**Affiliations:** I Second-year resident in Clinical Oncology, Hospital São Lucas, Pontifícia Universidade Católica do Rio Grande do Sul (PUCRS), Porto Alegre, Rio Grande do Sul, Brazil.; II Medical student, Pontifícia Universidade Católica do Rio Grande do Sul (PUCRS), Porto Alegre, Rio Grande do Sul, Brazil.; III MD, PhD. Head of Oncology, Hospital São Lucas, Pontifícia Universidade Católica do Rio Grande do Sul (PUCRS), Porto Alegre, Rio Grande do Sul, Brazil.

**Keywords:** Antibodies, neoplasm, Antibodies, monoclonal, Lymphoma, Lymphoma, B-cell, marginal zone, Hematologic neoplasms, Anticorpos antineoplásicos, Anticorpos monoclonais, Linfoma, Linfoma de zona marginal tipo células B, Neoplasias hematológicas

## Abstract

**CONTEXT:**

Splenic marginal zone lymphoma (SMZL) is a lymphoproliferative B-cell disorder that has a favorable prognosis, with estimated overall five-year survival of 70%. The majority of symptomatic patients undergo splenectomy, while a few receive first-line chemotherapy, especially with purine analogues. There are no specific treatment guidelines for patients for whom splenectomy fails to provide a cure. It is still unclear whether these patients should undergo cytotoxic chemotherapy, considering they have now a relapsed lymphoma (which is theoretically more aggressive), or whether they should be spared from treatments of greater toxicity, given that their disease usually develops with a more indolent course, even when it recurs.

**CASE REPORT:**

Here, we present two patients whose disease recurred after splenectomy and for whom rituximab monotherapy provided satisfactory treatment. From these cases, it can be suggested that postponement of cytotoxic treatments may be possible in at least some situations. It needs to be emphasized that the evidence to support this approach is based only on case reports, since there are no randomized clinical trials on this subject.

## RESUMO

**CONTEXTO:**

Os linfomas da zona marginal esplênicos constituem uma desordem linfoproliferativa de células B que apresenta um prognóstico favorável, com sobrevida global de cinco anos estimada em 70%. A maioria dos pacientes sintomáticos é submetida a esplenectomia enquanto alguns recebem quimioterapia terapêutica de primeira linha, especialmente com análogos de purinas. Não existem diretrizes específicas para o tratamento dos pacientes que falham à esplenectomia: ainda é incerto se deveriam ser tratados com quimioterapia citotóxica, em virtude de apresentarem um linfoma recidivado (e teoricamente mais agressivo) ou se deveriam ser poupados de um tratamento mais tóxico pelo fato de apresentarem uma doença que usualmente se desenvolve de forma mais indolente, mesmo quando recidivada.

**RELATO DE CASO:**

Nesta publicação, são apresentados dois casos nos quais a doença recidivou após esplenectomia e que foram satisfatoriamente tratados com monoterapia com rituximabe. A observação desses casos sugere que a postergação de tratamentos citotóxicos pode ser possível pelo menos em algumas situações. Cabe ressaltar que a evidência para essa conduta é embasada apenas em relatos de caso, uma vez que não existem ensaios clínicos randomizados a respeito desse tema.

## INTRODUCTION

Splenic marginal zone lymphoma (SMZL) with or without villous lymphocytes belongs to the group of the marginal zone lymphomas. SMZL has a favorable outcome, with a overall five-year survival of around 70%. Because of the rarity of the disease, there is no established standard therapy. Patients with asymptomatic disease are generally managed using a watch-and-wait strategy, while the majority of symptomatic individuals undergo splenectomy (or splenic irradiation), and a few receive front-line chemotherapy, especially with purine analogues.^1^ Here, we describe two cases in which the response to rituximab was very effective after post-splenectomy recurrence.

## CLINICAL CASES

A previously healthy 41-year-old man was diagnosed with SMZL in April 1996 (immunophenotype of circulating lymphocytes that was compatible with SMZL with villous lymphocytes). The leukocyte count was 22,000, with 80% mature lymphocytes, and the platelet count was less than 10,000/mm^3^. The patient presented massive splenomegaly. No lymphadenopathy was found from computed tomography (CT) scans at this time. The bone marrow was not compromised. The patient was promptly subjected to splenectomy, which confirmed the diagnosis of SMZL infiltration. Three months after the procedure, the platelet count had risen to 80,000/mm^3^ and the leukocyte count had fallen to 15,000/mm^3^. In October 2000, the platelet count was found to have fallen again to under 10,000/mm^3^ and the leukocyte count had risen to 85,000/mm^3^, with 80% villous lymphocytes. This time, an inguinal mass was palpable. A biopsy revealed recurrence of the lymphoma. Because the patient refused to receive cytotoxic therapy, monotherapy with rituximab was proposed. The patient received 375 mg/m^2^, for each of the four cycles every 28 days. The patient achieved complete remission after the fourth cycle. Two further cycles of rituximab were administered, three and six months after remission. In July 2005, the patient presented a mass in the lumbar area, a rapid fall in the platelet count and lymphocytosis. The mass was biopsied, and was again shown to be composed of villous lymphoma cells. A new course of rituximab was administered, using the same schedule as before. The patient achieved partial remission at the end of the fourth cycle and was found to be maintaining a platelet count of over 100,000/mm^3^ and lymphocytosis of under 5,000/mm^3^ at his last consultation, in January 2010.

A 58-year-old woman was diagnosed with SMZL in April 2003, with enlarged lymph nodes in the left cervical region. A chest CT scan revealed mediastinal enlargement. No other lymphadenopathy was observed in other scans. She had slight splenomegaly. Her leukocyte count was 16,000/mm^3^, with 10,000 lymphocytes/mm^3^. The immunophenotype was compatible with SMZL with villous lymphocytes. The bone marrow was not compromised. The patient underwent splenectomy and sustained complete remission for 13 months. In May 2004, her lymphocyte count started to rise and most of the circulating cell population was composed of villous lymphocytes. The mediastinal mass and lymphadenopathy recurred. At this time, the patient was referred to our center, and we decided to avoid chemotherapy and start rituximab as a single agent, because she presented with a creatinine level slightly over the normal range. She received the monoclonal antibody following the same schedule as used for our other patient, described above. After the fourth cycle of rituximab, she had achieved complete remission. A flow cytometry analysis performed in December 2007 revealed a persistent SMZL clone, but currently, the patient remains without any clinical signs of the lymphoma, more than five years after receiving rituximab.

## DISCUSSION

For management of symptomatic SMZL, splenectomy is still considered to be the front-line treatment of choice, since this treatment has shown survival advantage over chemotherapy. The overall five-year survival after splenectomy is estimated to be 71%^2^ and the time that elapses until progression has ranged from six months to seven years (average of four years).^1^

Patients whose condition progresses even after splenectomy, or those who have a more aggressive tumor, may be successfully managed with chemotherapy, using alkylating agents or purine analogues. However, considering the indolent course of SMZL, the therapeutic decision should be directed towards less toxic agents. In this setting, because of the low toxicity profile of rituximab, this monoclonal antibody has emerged as a convenient alternative for treating patients who refuse to undergo splenectomy or who are clinically unfit for a surgical procedure. Bennett et al. published a series with 11 patients treated with rituximab as second-line treatment, after failure of alkylating agents. Ten of the 11 patients responded to rituximab and five of them remained progression-free after a median follow-up of 58 months. The median clinical remission interval was 46 months.^3^ Kalpadakis et al. published a series in which 16 patients were treated with rituximab as first-line therapy. All patients had an objective response and 11 achieved a complete response. Twelve patients received maintenance rituximab therapy and 11 of them had no evidence of progression after a median follow-up of 28 months.^4^

Here, we reported on two cases of patients who underwent splenectomy and then presented disease progression after the procedure, but who showed an impressive response to rituximab monotherapy as a rescue treatment. Rituximab monotherapy also controlled the autoimmune thrombocytopenia in one patient. Fabri et al. reported a case in which rituximab resolved autoimmune hemolytic anemia.^5^ Moreover, we found that rituximab was also effective when used to treat a relapsing patient who had previously used the drug.

The role of rituximab as maintenance therapy, in order to prolong the time that elapses until progression occurs, still needs to be confirmed. Both patients in the present report received further doses of rituximab with an apparent effect on disease control. Nevertheless, these observations need to be validated with studies containing a larger group of patients. Bennett and Schechter recently published an analysis on a retrospective series of 52 patients in which rituximab as monotherapy seemed to be a good alternative to splenectomy, achieving overall responses of 88% to 100%, but their findings go beyond the scope of the present paper, since our patients were treated because of recurrence after splenectomy.^6^

Considering the lack of established data regarding this rare and clinically challenging issue, we made a systematic search for indexed articles published on this topic. In order to make our search as wide as possible, we did not apply any limits regarding the date of publication, the language used or the type of article. From our search in the PubMed database using the terms “((rituximab) AND marginal zone B cell lymphoma) AND therapy”, we found 163 papers, of which six were related to the current topic. We also searched in other databases, such as Lilacs (Literatura Latino-Americana e do Caribe em Ciências da Saúde) and the Cochrane Library, but no articles relating to this topic were found. It should be noted that a broader search strategy was used in relation to these last two databases (Table 1).

**Table 1.** Total numbers of papers indexed by Lilacs (Literatura Latino Americana e do Caribe em Ciências da Saúde), IBECS (Índice Bibliográfico Espanhol em Ciências da Saúde), Medline and the Cochrane Library with regard to the topic of rituximab as monotherapy for cases of splenic marginal zone lymphoma

Platform	Searching terms	Results
PubMed	((rituximab) AND marginal zone B cell lymphoma) AND therapy	2 reviews
		1 case report
		3 case series
Lilacs	rituximab AND lymphoma AND therapy	None
IBECS	rituximab AND lymphoma AND therapy	None
Cochrane	rituximab AND lymphoma AND therapy	None

## CONCLUSIONS

The way in which cases of recurrence of splenic marginal zone lymphoma after splenectomy should be managed is still not fully established. Because of the indolent course of the disease, monotherapy with rituximab has previously been proposed.^3,4^ In some cases, the need for a more aggressive form of therapy is suggested by the presence of comorbidities such as autoimmune disorders. The cases that we presented here allowed us to observe that even in these situations, the use of rituximab monotherapy might be a valid option when postponement of cytotoxic chemotherapy is an issue. Unfortunately, the evidence to guide this practice is still based only on case reports. More scientifically rigorous evidence is necessary to make this the standard of care in these cases, since there are no clinical trials on this subject.
